# Dynamic Contrast-Enhanced and Diffusion-Weighted Magnetic Resonance Imaging Noninvasive Evaluation of Vascular Disrupting Treatment on Rabbit Liver Tumors

**DOI:** 10.1371/journal.pone.0082649

**Published:** 2013-12-23

**Authors:** Haibo Shao, Yicheng Ni, Jian Zhang, Feng Chen, Xu Dai, Guoguang Fan, Ziping Sun, Ke Xu

**Affiliations:** 1 Department of Radiology, the First Affiliated Hospital of China Medical University, Shenyang, China; 2 Section of Radiology, Department of Medical Diagnostic Science, University of Leuven, Leuven, Belgium; 3 Plant Resources and Chemistry of Traditional Chinese Medicine, Jiangsu Provincial Academy of Traditional Chinese Medicine, Nanjing, China; 4 Radiation Medical Institute, Shandong Academy of Medical Sciences, Jinan, China; Vanderbilt University, United States of America

## Abstract

Evaluation of vascular disrupting treatment (VDT) is generally based on tumor size and enhancement on conventional magnetic resonance imaging (MRI) which, unfortunately, may be limited in providing satisfactory information. The purpose of the study is to evaluate consecutive changes of 20 rabbit VX2 liver tumors after VDT by dynamic contrast-enhanced MRI (DCE-MRI) and diffusion-weighted imaging (DWI) at a 3.0 T MR unit. Twenty four hours after intravenous injection of Combretastatin A-4-phosphate (CA4P) at 20 mg/kg, DCE-MRI derived Maximum Slope of Increase (MSI) and Positive Enhancement Integral (PEI) decreased sharply due to sudden shutting down of tumor feeding vessels. DWI derived Apparent Diffusion Coefficient (ADC) in tumor periphery decreased because of ischemic cell edema. On day 4, an increase of MSI was probably caused by the recovery of blood supply. A remarkable increase of ADC represented a large scale of necrosis among tumors. On day 8, the blood perfusion further decreased and the extent of necrosis further increased, reflected by lower MSI and PEI values and higher ADC value. On day 12, a second decrease of ADC was noticed because the re-growth of periphery tumor. The experimental data indicate that the therapeutic effects of VDT may be noninvasively monitored with DCE-MRI (reflecting tumor blood perfusion) and DWI (reflecting the changes of histology), which provide powerful measures for assessment of anticancer treatments.

## Introduction

Tumor vascular targeting strategies can be divided into two different approaches. Angiogenesis inhibitors (AIs) seek to inhibit the tumor-initiated angiogenic process by interrupting essential aspects of angiogenesis to prevent new blood vessel formation [1]–[3]. An alternative approach involves the application of therapeutics seeking the preferential destruction of the established tumor vessel network [4], [5]. These vascular disrupting agents (VDAs) cause direct damage to the previously established tumor endothelium, resulting in a rapid and selective vascular shutdown and secondary tumor cell death caused by ischemia [6]–[10].

Combretastatin A-4-phosphate (CA4P) is a representative VDA that has undergone several preclinical and clinical studies [11]–[17]. Evaluation of tumor response by imaging in these studies is generally based on tumor size and enhancement on conventional magnetic resonance imaging (MRI). Unfortunately, imaging techniques may be limited in providing clinically satisfactory information about dynamic changes based on blood perfusion and extent of necrosis. Improvements of current imaging techniques play a critical role in finding the optimal strategy to determine treatment success and guide future therapy [18]–[20].

Recently, advanced MRI methods, including dynamic contrast-enhanced MRI (DCE-MRI) and diffusion-weighted imaging (DWI), have been used in the evaluation of treatment response [21]–[24]. DCE-MRI techniques are able to characterize the microvascular response of tumors to vascular disrupting therapies. DWI techniques are used to evaluate the extent of tumor necrosis and to assess tumor response after therapy by detecting the thermally induced random movement of water molecules in biologic tissues.

In this study, we employed animal liver tumor models because they are more popular than those in other organs. The liver is the largest internal organ that not only generates various primary tumors of hepatic origin, such as hepatocellular carcinoma and cholangiocarcinoma, but also harbors secondary tumors, e.g., liver metastasis of colorectal cancer, through its abundant blood supply and lymph drainage. Furthermore, distinguished from rodent liver tumor models in previous studies, we used rabbit VX2 liver tumor models to evaluate the role of DCE-MRI and DWI in determining consecutive changes after intravenous administration of CA4P, which had never been reported.

## Materials and Methods

### Ethics Statement

Animal care followed the Chinese Community Standard for care and use of laboratory animals, and the protocols for animal experimentation were approved by the institutional Animal Care and Use Committee of China Medical University. All surgery was performed under sodium pentobarbital anesthesia, and all efforts were made to minimize suffering.

### Animal Model

New Zealand white rabbits (Laboratory Animal Use license: SYXK 2008-0005) were provided by the institutional laboratory animal center. Male and female rabbits weighed between 2.5 to 3.5 kg. The VX2 tumor blocks were taken out of liquid nitrogen, resuscitated in 37°C water bath for 10 minutes, and minced into cell suspension, which was then injected into the hind legs of 2 carrier rabbits and grown for 2 weeks. Resultant tumors were then harvested from the carriers and were cut into small blocks with a diameter of 1 mm. For rabbits receiving VX2 tumor implantation, 3% soluble pentobarbitone (0.5 mg/kg, Sigma) was administered intramuscularly. Abdominal skin of each recipient rabbit was shaved and disinfected with ethanol and povidine iodine. Then an 18-gauge coaxial trocar was inserted percutaneously into the left lobe of the liver using CT-guided method. A tumor block was pushed into the tip of the trocar by a priming wire and embedded in the liver. The tumor blocks were allowed to grow in the rabbit livers for 2 weeks to develop solitary lesions with a diameter of approximately 2 centimeters.

### Study Protocol

Twenty qualified liver tumor models were enrolled in this study. CA4P was dissolved in 2 ml sterile saline and given via marginal ear vein as single injection at a dose of 20 mg/kg. MRI scan were performed in all rabbits before (baseline) and at 24 hours, 4 days, 8 days and 12 days after CA4P treatment. For postmortem verification of the imaging findings, 2 rabbits were sacrificed at each time point immediately after MR scan.

### MR Imaging

MRI was performed at a 3.0 T MR unit (Signa HDx, General Electric Medical Systems, USA) with a maximum gradient strength of 40 mT/m equipped with a HD Quadrature Knee/Foot Coil. The rabbits were anesthetized with intramuscular injection of 3% soluble pentobarbitone (1.0 mg/kg; Sigma). To keep a symmetrical supine position, the rabbits were fixed on a home-made plastic holder which was matched with the knee coil in diameter.

Sagittal, coronal, and axial pilot images were first obtained to localize subsequent MRI acquisitions. For each imaging sequence except DCE-MRI, 18 axial images were obtained with a section thickness of 3.0 mm and an intersection gap of 1.0 mm. All sequences were performed with the same geometry to maintain comparability between the different imaging sequences.

For fast spin-echo (FSE) T2WI: repetition time (TR)/echo time (TE) of 5120/85 ms; field of view (FOV) of 160×160 mm; imaging acquisition matrix of 256×192; and total acquisition time of 2 minutes 24 seconds.

For spin-echo (SE) T1WI: TR/TE of 200/5.8 ms; FOV of 160×160 mm; imaging acquisition matrix of 256×192; and total acquisition time of 3 minutes 1 second.

DCE-MRI was performed using a 3-dimensional T1-weighted gradient-echo sequence with fat saturation: TR/TE of 5/2.1 ms; FOV of 160×160 mm; imaging acquisition matrix of 272×160; and total acquisition time of 1 minute 57 seconds. In total, 60 measurements were acquired. An intravenous bolus of gadodiamide (GE Healthcare AS) was administered with a manual injection at a dose of 0.1 mmol/kg after the first 12 measurements.

DWI was acquired with a 2-dimensional SE echo-planar imaging sequence: TR/TE of 5000/77.3 ms; FOV of 160×160 mm; imaging acquisition matrix of 128×128; b values of 0 and 1000 mm^2^/s along axial direction; and acquisition time of 41 seconds.

Contrast enhanced T1 weighed images (CE-T1WI) were acquired after intravenous bolus injection of gadodiamide at 0.2 mmol/kg.

### Image Analysis

The acquired images were sent to a dedicated workstation (Advantage Workstation, ADW 4.5, GE Medical Systems) and analyzed by two experienced radiologists.

The tumor volume was calculated on the basis of fast spin-echo T2WI. By using an operator-defined region of interest (ROI), the area of tumor was manually delineated on each tumor-containing slice. The total area of tumor for the slices was calculated after summation and then multiplied by the slice thickness plus gap to obtain the total tumor volume, see formula (1). The doubling time (DT) of tumor volume was calculated with formula (2), where T–T0 indicates the length of time between two measurements, and V0 and V denote the tumor volume at two consecutive points of measurement.
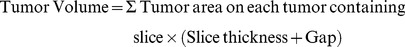
(1)

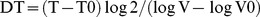
(2)


The DCE images were analyzed using the FuncTool (version 4.5.5). The signal range was adjusted to clearly show both liver and tumor. According to the parameter selection method described previously, maximum slope of increase (MSI) and positive enhancement integral (PEI) were selected as the parameters. The corresponding pseudo-color images were automatically generated. Based on the original image, a ROI was delineated along the tumor on the central slice with a free-hand tool. The value of corresponding parameter of the whole tumor was calculated automatically. The values of MSI and PEI of liver were generated by placing circular ROIs containing at least 40 pixels on corresponding locations in central slices.

The DW images were also analyzed using the FuncTools in the workstation. Apparent diffusion coefficient (ADC) maps were generated automatically from the DW images, and values were calculated by placing a ROI on ADC maps. To calculate the ADC of the entire tumor, the tumor was delineated with an operator-defined ROI for each tumor-containing slice. The corresponding ADC values were summed and then averaged using formula (3). To calculate the ADC of the periphery and of the center of the tumor, three circular ROIs containing at least 40 pixels were placed in the periphery as well as in the center of the tumor on the central tumor-containing slice, and the mean value was calculated as the ultimate ADC value. Similarly, three circular ROIs were placed on the livers surrounding the tumor to calculate the ADC of the livers.
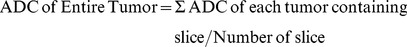
(3)


### Histological Analysis

The rabbits were sacrificed immediately after MR scan by intravenous injection of 10 ml of 10% kalium chloratum. After fixation in 10% formaldehyde solution, the tumors harvested from the rabbit livers were sliced at approximately 5 mm intervals in the axial plane corresponding with the MR images. The regions of interest were resected from the histological slices and embedded in paraffin. Then, 3 µm sections were stained with hematoxylin-eosin (HE), which were evaluated by an experienced pathologist. The sections were visualized with an Olympus CH30 Microscope and assessed for viable tumor cells, interstitium and necrosis with magnification of ×100 to ×400. Thereafter, the histological sections were compared visually with the corresponding conventional images and the functional maps.

### Statistical Analysis

Statistical analysis was performed by using Graphpad Prism 5.0 software demo. Numerical data are reported as the mean ± standard deviation. For statistical comparison of values of functional parameters obtained at two consecutive time points, unpaired two-tailed Student *t* tests were performed. Results were considered significant at p<0.05.

## Results

### General Conditions

Rabbit liver tumor models were successfully established for this experiment. All animals survived the surgical procedures, drug injection, tumor growth and in vivo imaging sessions except one which died of anesthesia overdose during the last MR imaging session.

### Tumor Growth

**Figure 1:**
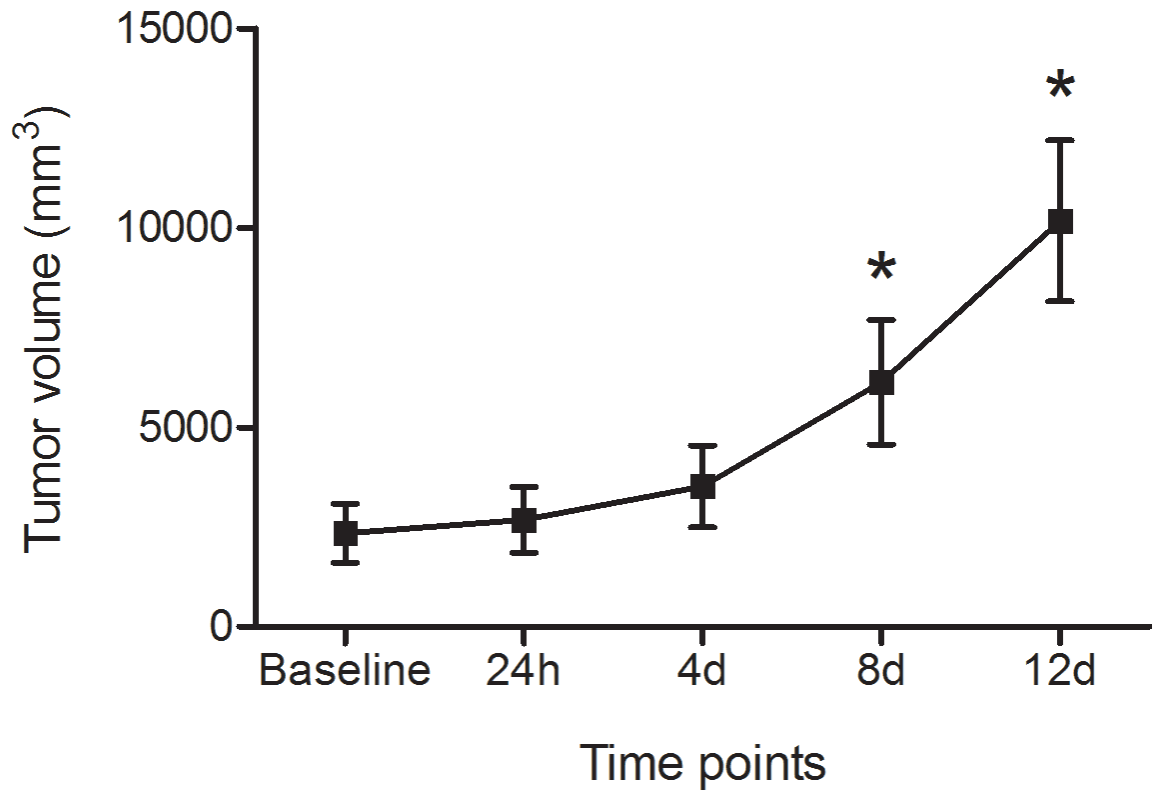
Tumor volume at different time points. Tumor volume was showed as Mean±SD. Because a couple of animals were sacrificed at each time point, the number of the animals was 20, 18, 16, 14, 12 respectively at the five time points from baseline to 12 d. The tumor grew gradually with time. Initially, the tumor grew slowly but accelerated since 4 d. The mean tumor volume was significantly larger than that at previous time point at 8 d and 12 d. *P<0.05.

The average tumor volume was 2344±739 mm^3^ at baseline. The tumors grew quickly, as shown in Figure 1. The average volume of tumors was 2681±824, 3514±1024, 6134±1563 and 10170±2019 mm^3^ at 24 h, 4 d, 8 d and 12 d, respectively. The mean tumor volume at 8 d and 12 d was significantly larger than that at previous time points (p<0.05, Figure 1). Tumor doubling time was 6.31±2.67 days during the 12 days.

### Conventional MR Images

**Figure 2:**
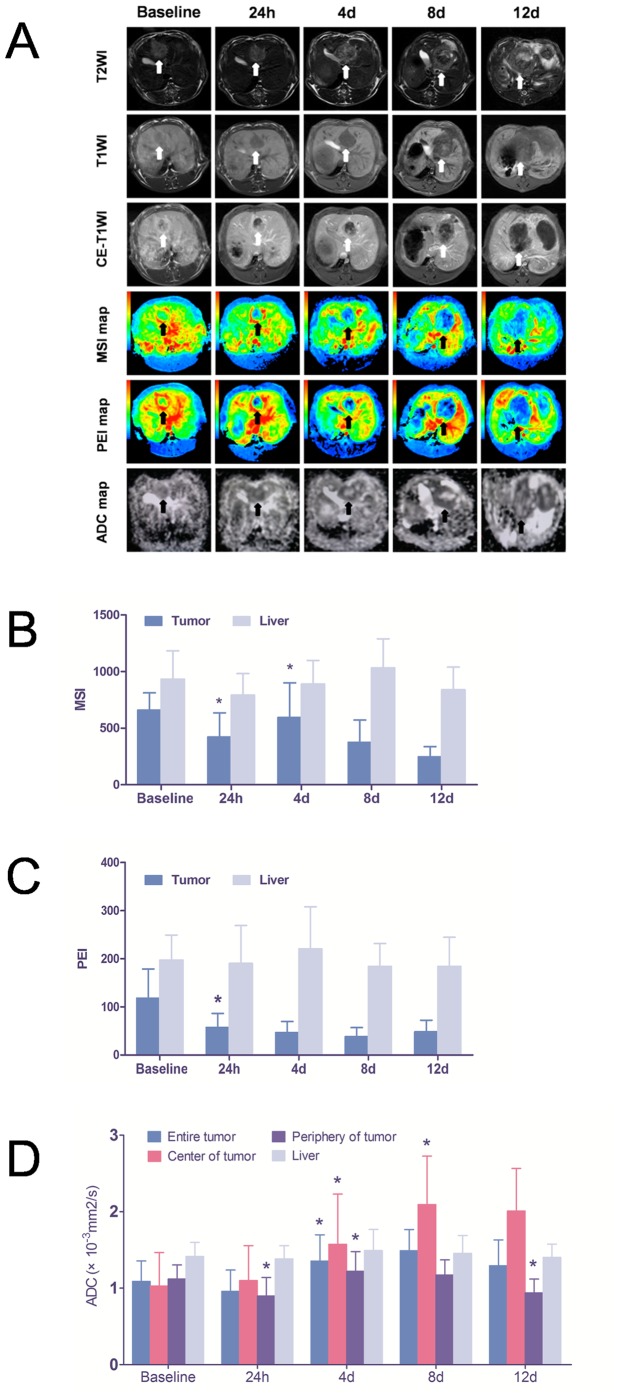
Conventional MRI, DWI and DCE MRI at different time points. (a) Representative T1WI, T2WI, CE-T1WI, DWI-derived ADC map and DCE MRI-derived MSI and PEI maps were enumerated in the figures. Twenty four hours after CA4P treatment, the enhancement of tumor was sharply decreased but the ADC map was similar to that at baseline. An obvious hyperintense center was noticed at day 4 and day 8 with only a thin hypointense peritumoral rim. On T1CE images, at same time, there was still no enhancement in the center, but a progressively enhanced rim was observed. At day 12, inhomogeneous enhancement was seen on the entire tumor with irregular nodules from the rim towards the center on both CE-T1WI and ADC map. The blood perfusion of tumor sharply decreased at 24 h because the shut-down of the tumor vessels on both MSI and PSI maps at 24 h, and gradual decrease was also observed at afterward time points. However, a recovery of blood perfusion on MSI map at 4 d might be due to the re-growth of tumor cells on the periphery. (b, c) Quantitative MSI (b) and PEI (c) values of tumor and liver were showed and compared with bar charts. (d) Quantitative ADC values of entire tumor, center or periphery of tumor, and liver were showed and compared with a bar chart. *The values had statistical changes compared with those at previous time point (P<0.05).

At baseline, the tumors showed a spherical, oval or spindle shape with a clear border easily demarcated from the surrounding liver with all imaging sequences. The tumor appeared hyperintense on T2WI and hypo-intense on T1WI. After injection of contrast agent, the tumors were enhanced. After administration of CA4P at the different time points, conspicuous changes were noticed on CE-T1WI images (Figure 2A). Twenty-four hours later, enhancement was sharply decreased. In most cases, thin enhanced rings around the tumor appeared at day 4 and became thicker at day 8 on CE-T1WI. Nodules from the enhanced rings oriented toward the unenhanced center were noticed in some cases at the later time points (Figure 2A). Meanwhile, the center of tumor was not enhanced at all follow-up time points until day 12 when some irregular enhanced regions appeared (Figure 2A).

### Dynamic Contrast-enhanced MR Images

The continuous changes of tumor blood perfusion were shown by CE-T1WI images and corresponding MSI and PEI map in figure 2A. The corresponding quantitative values of MSI and PEI are shown in Figure 2B,C and table 1,2. At baseline the tumor showed abundant blood perfusion on PEI map and MSI map especially on the periphery of tumor. Twenty-four hours after administration of CA4P, blood perfusion of the tumor was sharply decreased on both MSI (P<0.05) and PEI (P<0.05) maps. Blood perfusion gradually decreased on both MSI and PEI maps with tumor growth, although there was no statistically significant difference in quantitative values between continuous two time points except an increase of MSI from 24 h to 4 d (P<0.05).

**Table 1:** MSI values of tumor and liver at different time points.

	Baseline	24 h	4 d	8 d	12 d
Tumor	669±153	424±212*	596±304*	375±197	247±90
Liver	933±250	792±191	889±210	1033±257	839±201

P<0.05.

**Table 2:** PEI values of tumor and liver at different time points.

	Baseline	24 h	4 d	8 d	12 d
Tumor	118±61	57±29*	46±23	38±19	48±24
Liver	197±52	190±79	220±88	184±47	184±61

P<0.05.

### Diffusion-weighted MR Images

The tumors appeared hypointense on DWI-derived ADC maps at baseline. The corresponding ADC values for the entire tumor, tumor center, tumor periphery and liver are shown in figure 2 and table 3. Changes were noticed on DWI images with the growth of the tumor at different time points (Figure 2A, D). Twenty-four hours after administration of CA4P, the ADC maps were similar in appearance to those obtained before drug administration (Figure 2A). The corresponding ADC values (Figure 2D and table 3) showed no significant differences except a slight decrease in the periphery of tumor (P<0.05 vs. baseline). At day 4 and day 8, obvious large-scale changes of hyperintense signals appeared in the center of the tumor (Figure 2A). Compared with measurements at the 24-hour time point, the mean ADC values increased significantly in both the center and peripheral regions at day 4, leading to an increase in ADC in the entire tumor (Figure 2D and table 3). The mean ADC values increased progressively in the center at day 8, but the change in ADC ceased in both the peripheral and central tumors (Figure 2D and table 3). The mean ADC decreased in the periphery at the 12-day point, leading to a decrease in ADC in the entire tumor, although the ADC values had no significant changes in the center (Figure 2D and table 3). The mean ADC of the surrounding liver did not show any significant changes during the entire follow-up period (Figure 2D and table 3).

**Table 3:** ADC values (mm^2^/s) of tumor and liver at different time points.

	Baseline	24 h	4 d	8 d	12 d
Entire tumor	1.09±0.27	0.96±0.30	1.35±0.34*	1.49±0.28	1.29±0.34
Peripheryof tumor	1.12±0.19	0.90±0.24*	1.22±0.26*	1.17±0.20	0.94±0.18*
Centerof tumor	1.03±0.48	1.10±0.46	1.57±0.66*	2.09±0.63*	2.01±0.56
Liver	1.42±0.18	1.38±0.17	1.49±0.27	1.46±0.23	1.40±0.17

P<0.05.

### Histopathologic Findings

**Figure 3:**
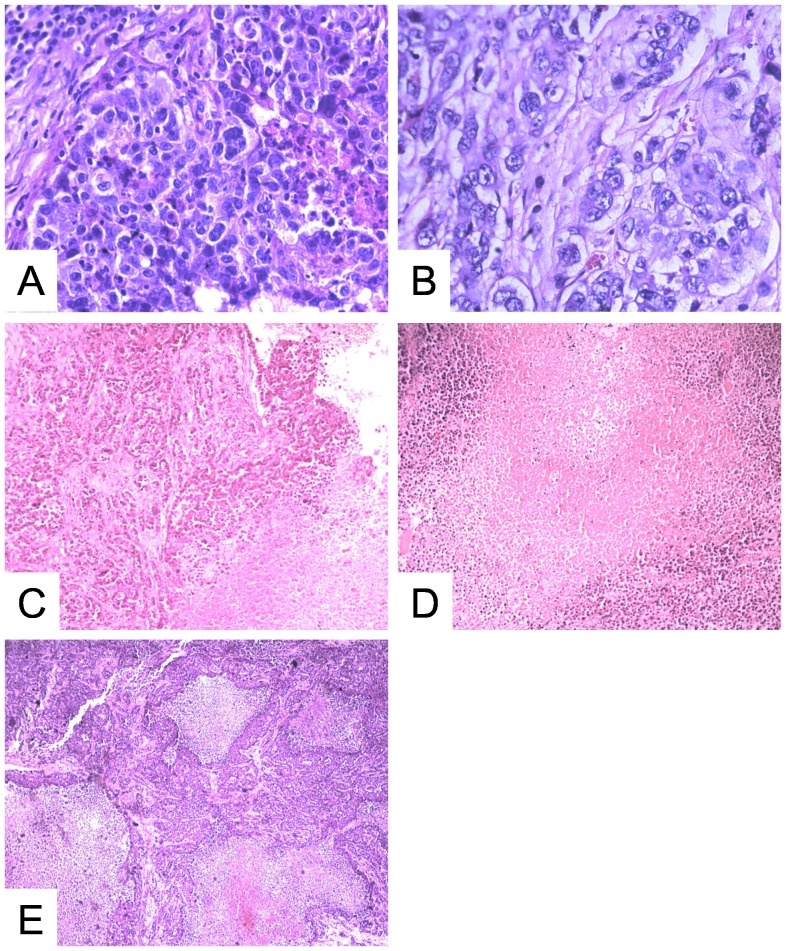
Histology under microscope with H&E staining. (a) VX2 carcinoma cells rich in mitosis were observed at baseline. (b) Twenty-four hours after administration of CA4P, although the tumor cells experienced heavy edema, the membranes of most tumor cells were still intact. (c, d) At 4 d and 8 d, H&E sections showed large thorough necrotic area, which appeared unanimously eosinophilic. (e) Central necrosis mixed with re-growing tumor cells were seen in the outer peripheral rims. *400× for (a) and (b); 100× for (c), (d) and (e).

Histology showed VX2 carcinoma cells rich in mitosis with central irregular necrosis at baseline (Figure 3A). Twenty-four hours after administration of CA4P, although the tumor cells experienced heavy edema, the membranes of most tumor cells were still intact (Figure 3B). At 4 days and 8 days, HE sections showed a large, thoroughly necrotic area, which appeared eosinophilic (Figure 3C, D). Viable tumor cells were seen only in the outer peripheral rims. The nodules shown on MR images were verified as re-growing tumor tissue via histology. The irregular enhanced regions appearing in the center of tumors at 12 d were verified as central necrosis mixed with re-growing tumor via histology (Figure 3E).

## Discussion

The present study has unambiguously demonstrated that continuous changes in rabbit VX2 liver tumors after the treatment of vascular disrupting agent CA4P can be monitored using a clinically available 3.0-T MRI scanner. MRI is currently the most versatile method for noninvasive assessment of malignant tumor responses to therapy [18]–[26]. It is realized that a comprehensive array of MRI-derived parameters have been used as morphologic, physiological, and metabolic/molecular biomarkers for detection, characterization, and therapeutic evaluation of malignancies [27]–[29]. Multiparametric functional MRI was employed in this study to quantitatively evaluate intratumoral therapeutic events which were demonstrated by postmortem histopathology.

Rabbit VX2 liver tumors are the most commonly used large animal models to imitate primary or secondary liver cancers in humans [30]–[33]. Recently, several studies had reported the functional MRI characteristics of experimental liver tumors treated by CA4P in rat and mouse models, with similar results to the present study [20], [24], [27], [34], [35]. To our knowledge, this study is the first time that a liver tumor model in large animals has been used to evaluate the dynamic characteristics of vascular disrupting treatment using functional MRI.

DCE-MRI and DWI are newly developed, noninvasive imaging techniques for monitoring the effectiveness of a variety of treatments, including chemotherapy, hormonal manipulation, radiation therapy, and drug therapy with new antiangiogenic agents and vascular targeting agents [27], [36], [37]. DCE-MRI may provide functional information about vascularity, blood perfusion and vascular permeability. MSI and PEI are selected parameters that reflect changes in blood perfusion or vascular permeability. DWI has been used to differentiate between viable and necrotic tumor tissue in animal tumor models. This MR imaging technique enables the depiction of molecular diffusion, which is the Brownian motion of water protons in biologic tissues [38]. The calculation of the ADC allows for the quantification of this motion.

As presented in the study, 24 hrs after treatment with CA4P, conspicuous changes appeared on DCE-MRI. Both MSI and PEI maps showed sharp decreases in blood perfusion both in the center and periphery of the tumor; quantitative values showed statistical difference from baseline measures. However, no obvious changes were found on DWI-derived ADC maps except a decrease in the periphery of the tumor. Microscopic findings showed tumor cell edema and loss of connection between cells caused by ischemia, especially in peripheral regions. This is likely the cause for the decrease in ADC. Experiencing a short period of ischemia, Na+,K+-ATPase pump in the membrane of tumor cell lost energy and the concentration of K+ increased in the interstitial space, which led to the inflow of hydrogen proton directly [39]. At this time, the cytomembrane was still intact which restricted the movement of more water protons. The free water molecules were relatively reduced in interstitial space, hence decreased ADC values in the tumor. Therefore, DCE-MRI quantitatively reflected blockage of blood flow to the tumor, while DWI reflected earlier changes in histology at both the cellular and subcellular scales.

At 4 days, when intracellular edema increased, the cellular membrane was broken down and complete necrosis of tumor appeared. Meanwhile, a thin ring of viable tumor cells were noticed, which may lead to an increase in blood supply in the periphery of the tumor, reflected by MSI. The ADC value increased because of an increased flow of water molecules in interstitial space. A large hyperintense area appeared in the tumor center with only a thin hypointense rim remaining in the periphery on the ADC map. The histological findings showed cytolysis of tumor cells in the center but intact and viable tumor cells at the periphery. Due to progressive necrosis, the ADC value became higher at day 8. However, we also observed that the viable tumor rim became thicker, leading to increased tumor volume. Indeed, a rim of viable cells at the periphery is often observed after treatment with CA4P [20], [24], [27]. There is a tendency of blood flow to recover more quickly in the peripheral than central regions of the tumor [40]. The response of the peripheral vessels to CA4P treatment appears weaker relative to those in the center, although the exact mechanism for this phenomenon is unclear. This limits the effectiveness of CA4P, causing rapid re-growth of tumors after treatment [41]. Accordingly, we observed an increase in tumor volume and a decrease in ADC due to rapid re-growth in the rim of tumor.

To deal with the rapidly growing rim of the tumor and to improve therapeutic effects, measures such as adjuvant conventional cytotoxic approaches or radiation therapies were taken [42]. Also, repeated drug administration at time intervals seemed more effective, which could be accurately reflected by DCE-MRI and DWI, as supported by the histological changes [43]. Recently, we used a necrosis avid agent hypericin labeled with radioactive iodine-131 to target the necrosis induced by CA4P. The tumor growth was well controlled and was able to be clearly monitored with functional MRI [34], [35], [44].

This study was limited by the use of VX2 tumors, which are of non-hepatic origin. However, this tumor type is a convenient model for the study of liver cancer in the animal because of the similarities in blood supply, genotype, and metabolism to advanced human hepatic carcinoma. Furthermore, abdominal respiratory movement in rabbits could not be completely eliminated during MRI. Movement continued to cause some variations in the MRI measurements. Anatomic distortion was present in the parameter derived functional maps when compared with conventional images, which was caused by susceptibility effects of the 3 T MR unit. Third, although by using DCE-MRI with clinically available software we were able to quantitatively measure the blood perfusion-related parameters, which are particularly crucial for studying VTAs, the accuracy of the method was not validated by other more delicate in vivo techniques such as Doppler ultrasound and direct flowmetric measurement. Further immunohistochemical staining such as microvessel staining which may reflect blood perfusion parameters of DCE-MRI were not performed. These shortcomings will be addressed in our ongoing research.

## Conclusion

CA4P proves to be an effective VTA to cause rapid vascular shutdown and severe necrosis in rabbit VX2 liver tumors, although recurrence appeared early at the periphery. The therapeutic effects of CA4P can be noninvasively monitored with functional MRI. DCE-MRI and DWI sequences provide potent measures for assessment of anticancer treatment.
